# Editorial: Accelerating to 2030 - doubling down on HIV prevention to end HIV/AIDS as a public health threat

**DOI:** 10.3389/frph.2025.1670024

**Published:** 2025-08-29

**Authors:** Robyn Eakle, Jason Reed, Nittaya Phanuphak, Kenneth Ngure, Kimberly E. Green

**Affiliations:** ^1^ Independent Researcher, Alexandria, VA, United States; ^2^ Independent Researcher, New Orleans, LA, United States; ^3^ Institute of HIV Research and Innovation, Bangkok, Thailand; ^4^ School of Public Health, Jomo Kenyatta University of Agriculture and Technology, Nairobi, Kenya; ^5^ Department of Global Health, University of Washington, Seattle, WA, United States; ^6^ PATH, Seattle, United States

**Keywords:** HIV prevention, PrEP, product choice, innovation, improving service delivery

**Editorial on the Research Topic**
Accelerating to 2030 – doubling down on HIV prevention to end HIV/AIDS as a public health threat

The global HIV programming landscape has changed dramatically since the inception of this Research Topic. In January 2025, the United States (US) halted nearly all foreign assistance funding, including for the President's Emergency Plan For AIDS Relief (PEPFAR), leaving countries all over the world scrambling to fill sudden gaps in critical services, especially for people living with HIV (PLHIV) (1). The unexpected halt in funding threatens millions of lives and creates significant risk in reversing the trajectory of new HIV infections which have been on a steady decline since 2010 (2). In addition to this most recent disruption, other countries have reduced their contribution to foreign assistance funding, including to organizations like the Global Fund for AIDS, Malaria, and Tuberculosis (Global Fund) (3).

HIV prevention has often been treated as an afterthought or “nice to have” within the larger context of HIV programming needs. However, programming and modelling data indicate that prevention and treatment are both needed to end HIV as a public health threat (4–6). This collection highlights the overall, ongoing imperative for prevention as well as specific programming and innovations to achieve goals and sustain gains.

Socio-political fallout impacting the global health landscape has generated even more need for innovation, creativity, and commitment to HIV prevention programming to rapidly turn off the tap of new infections and reduce medium to long-term costs. Ultimately, nine papers were included in this collection with unifying themes that emerged cutting across populations, interventions, and geographic regions. These themes highlighted the ongoing importance of person-centered care and interventions, local leadership and political will, and intentionally leveraging innovation to improve services. These elements can translate into powerful, successful programming to advance HIV prevention and more effectively curb epidemics.

The papers by Hamoonga et al., Naidoo et al., Tumusiime et al., Irie et al., and Nishimoto et al. articulate the importance of population specific programming for pregnant and breastfeeding women, adolescent girls and young women in Africa, black women in the US, and people who inject drugs (PWID), respectively. These papers feature vulnerable subpopulations within their epidemics and how addressing their needs can lead to improved, overall programming and outcomes. These examples underscore how improving access to tailored, people-centered services can advance broader, more effective and equitable access, which can be translated to many other contexts.

Milimu et al., Allinder et al., and Vu et al. demonstrated how local leadership and political will drove innovative programming in South Africa, Malawi, and Vietnam. In these papers, countries spearheaded systemic interventions to improve HIV prevention programming, particularly for pre-exposure prophylaxis (PrEP) scale-up. Differentiated services that allowed for access to PrEP outside of traditional clinic settings, service integration with sexual and reproductive health, and continuous evaluation of the services were key components that made for success. As illustrated by the papers, it was essential to include populations at the heart of the service design process to define what is important to them in each context - e.g., not every service and not all needs will look the same everywhere. Furthermore, sharing best practices and reflections contemporaneously allowed others to learn from and build upon these efforts. These systemic innovations are particularly timely and relevant now given the sudden reductions in funding further threatening the future of HIV prevention and could advance sustainable services.

Now, more than ever, innovation is crucial for current and future HIV prevention programming. New options like long-acting injectable PrEP and other formulations in the development pipeline, can improve uptake and effective coverage of HIV prevention overall and enhance service efficiency, especially if implemented through differentiated platforms (7). Expanded choice in prevention options was noted by Naidoo et al., Nishimoto et al., and Vu et al. as an important factor in improving PrEP uptake and effective use. These papers add to a growing body of literature showing that expanding prevention options along with service delivery points, improves overall use (7, 8). Long-acting injectables can provide lengthy, definitive protection (e.g., 2 months or 6 months), reduce visit frequency, and therefore alleviate burdens on service delivery points and systems.

Measurement of service impact and outcomes has long been a significant hurdle for HIV prevention programming (9). While HIV treatment clear goals such as the UNAIDS three 95's, HIV prevention has struggled to establish simple global metrics, further compounded by the difficulty in estimating the denominator of people in need of prevention (10, 11). The paper led by Mukherjee et al. contributes to the evolving literature assessing unmet need for PrEP, or the PrEP-to-need ratio (PnR). This analysis contributes to meaningful metrics for HIV prevention services to help better illustrate and define the importance of prevention interventions in overall epidemic control.

This collection fulfilled most of its aim to showcase important elements and an array of solutions for the future of HIV prevention. However, while the collection was open to all HIV prevention programming modalities, most submissions focused mostly on PrEP. Other aspects of HIV prevention programming such as condoms, voluntary medical male circumcision (VMMC), and more on long-acting injectables would have been welcomed. Additionally, new developments in service delivery, like expanding to private and community pharmacies, and data management through artificial intelligence (AI) (12–14), may now be the answers to advancing programming and establishing sustainable solutions going forward.

This collection brings together population and programming elements essential to the success of prevention in ending HIV as a public health threat. Many innovations in these papers were supported by Global Fund and/or PEPFAR, whose budgets are now being affected by massive reductions in funding, thus hampering the achievement of the 2030 goal. Prior to 2003 and the launch of PEPFAR, HIV had so severely impacted countries that mortality rates had affected entire economies (15–17). The world cannot afford to regress 20+ years. The transformation of the global HIV pandemic landscape since PEPFAR's inception has been so transformative that it is virtually impossible to remember HIV health outcomes from 2003. The world is much smaller and more interconnected than ever, such that parsimonious, isolationist political agendas may threaten global progress in health and development. Advancing tailored, innovative HIV prevention services that fit the specific needs of people will be key to preserving decades of advances, sustaining momentum, and averting setbacks culminating in the end of HIV as a public health threat.
